# Identifying differentially expressed proteins between amyloid positive and amyloid negative subjects based on Alamar multiplex assay data using MissionAD samples

**DOI:** 10.1002/alz70856_107031

**Published:** 2026-01-09

**Authors:** Pallavi Sachdev, Han Yin, Hongmei Niu, Larisa Reyderman, Michael C. Irizarry, David Verbel

**Affiliations:** ^1^ Eisai Inc., Nutley, NJ, USA

## Abstract

**Background:**

Single analyte blood‐based biomarkers such as *p*‐tau181 and *p*‐tau217 are promising biomarkers for identifying Alzheimer's disease (AD) pathology. However, multi‐analyte blood‐based biomarker panels are needed to further improve the detection, differential diagnosis, and screening of AD and to predict disease progression and assess response to therapy. The Alamar NULISASeq CNS Disease Panel (“NULISASeq”) simultaneously profiles 120 proteins associated with neurodegeneration, synaptic, and inflammatory pathways to support these goals. Multiplexed panels also maximize the potential of precious clinical trial biospecimens while also providing the sensitivity required to measure low abundant analytes.

**Methods:**

Plasma samples were collected during screening from a Phase 3 program for elenbecestat (MissionAD) in early AD and analyzed using NULISASeq. Data normalization was performed using the vendor protocol to form NULISA Protein Quantification (NPQ) units, used for statistical analysis. Analysis of Variance (ANOVA) was used to determine statistical significance of the difference in each target protein level between amyloid+ and amyloid‐ subjects as determined by PET visual read. *p*‐values were adjusted for multiplicity. Cohen's D was calculated to show the effect size of difference in target protein levels. The correlation of overlapping target protein levels between NULISASeq and other assays was evaluated.

**Results:**

A total of 124 subjects were included in the statistical analysis (74 amyloid+ and 50 amyloid‐). Statistical significance was observed in 7 plasma proteins (GFAP, MAPT, NEFH, SNAP25, pTau‐181, pTau‐217, pTau‐231) between amyloid+ and amyloid‐ subjects. pTau‐217 showed the largest effect size. In predicting amyloid status, using pTau‐217 alone also resulted in the highest AUC (0.87). Combining these proteins did not result in much improvement in prediction. NULISASeq protein levels of common ATN biomarkers showed high correlation with that of other assays in both plasma and CSF.

**Conclusion:**

Targeted multiplexed panels such as NULISASeq quantifies multiple analytes in a single run thereby reducing the amount of precious input material and the time required for analysis. This offers advantage over fluid and neuroimaging‐based measurements tailored to singular targets. We have demonstrated the utility of a multiplexed panel for reliable detection of amyloid status as well as highlighted the potential of discovering novel biomarkers associated with AD.

